# Suggestion for linkage of chromosome 1p35.2 and 3q28 to plasma adiponectin concentrations in the GOLDN Study

**DOI:** 10.1186/1471-2350-10-39

**Published:** 2009-05-09

**Authors:** Laura J Rasmussen-Torvik, James S Pankow, James M Peacock, Ingrid B Borecki, James E Hixson, Michael Y Tsai, Edmond K Kabagambe, Donna K Arnett

**Affiliations:** 1 Division of Epidemiology and Community Health, University of Minnesota School of Public Health, Minneapolis, MN, USA; 2 Heart Disease & Stroke Prevention Unit, Center for Health Promotion, Minnesota Department of Health, St Paul, MN, USA; 3 Division of Biostatistics, Washington University School of Medicine, Saint Louis, MO, USA; 4 Human Genetics Center, University of Texas-Houston School of Public Health, Houston, TX, USA; 5 Department of Lab Medicine and Pathology, University of Minnesota, Minneapolis, MN, USA; 6 Department of Epidemiology, University of Alabama at Birmingham School of Public Health, Birmingham, AL, USA

## Abstract

**Background:**

Adiponectin is inversely associated with obesity, insulin resistance, and atherosclerosis, but little is known about the genetic pathways that regulate the plasma level of this protein. To find novel genes that influence circulating levels of adiponectin, a genome-wide linkage scan was performed on plasma adiponectin concentrations before and after 3 weeks of treatment with fenofibrate (160 mg daily) in the Genetics of Lipid Lowering Drugs and Diet Network (GOLDN) Study. We studied Caucasian individuals (n = 1121) from 190 families in Utah and Minnesota. Of these, 859 individuals from 175 families had both baseline and post-fenofibrate treatment measurements for adiponectin. Plasma adiponectin concentrations were measured with an ELISA assay. All participants were typed for microsatellite markers included in the Marshfield Mammalian Genotyping Service marker set 12, which includes 407 markers spaced at approximately 10 cM regions across the genome. Variance components analysis was used to estimate heritability and to perform genome-wide scans. Adiponectin was adjusted for age, sex, and field center. Additional models also included BMI adjustment.

**Results:**

Baseline and post-fenofibrate adiponectin measurements were highly correlated (r = 0.95). Suggestive (LOD > 2) peaks were found on chromosomes 1p35.2 and 3q28 (near the location of the adiponectin gene).

**Conclusion:**

Two candidate genes, *IL22RA1 *and *IL28RA*, lie under the chromosome 1 peak; further analyses are needed to identify the specific genetic variants in this region that influence circulating adiponectin concentrations.

## Background

Adiponectin is an adipokine that is inversely related to both adiposity and many chronic disease risk factors in several populations. Adiponectin increases insulin sensitivity (i.e., the converse of insulin resistance) when administered intravenously to rats [1] and has decreased transcription in the visceral fat of obese as compared to lean humans [2]. In epidemiologic studies, adiponectin has been associated cross-sectionally with both waist or visceral adiposity [3,4] and euglycemic-clamp derived insulin sensitivity [5,6]. Adiponectin is also hypothesized to be protective in the pathogenesis of atherosclerosis [7,8], perhaps by reducing activity of iNOS in the vascular adventia [9] or by reducing accumulation of lipids in macrophage foam cells [10].

Given the relations of adiponectin with chronic disease risk factors, there is much interest in learning about the pathways through which adiponectin itself is regulated. Several studies have found circulating adiponectin to be heritable, with heritability estimates ranging from 0.42 to 0.93 [11-15], suggesting that genetic variants play a role in regulating adiponectin. To find these variants, several studies have performed whole-genome linkage scans to determine which areas of the genome may harbor genes influencing circulating adiponectin concentrations [12-16]. Unfortunately, likely because of the diverse study populations used in each of these scans, few significant linkage results have been replicated across studies.

The Genetics of Lipid Lowering Drugs and Diet Network (GOLDN) Study is a genetic family study that included a three-week trial of fenofibrate, a drug that significantly decreases triglycerides and increases high-density lipoproteins without increasing low-density lipoproteins [17]. Although the hypothesized pathway of action for fenofibrate does not involve adiponectin, two placebo-controlled studies have found that short trials of fenofibrate significantly increased circulating adiponectin [18,19], while two other short trials of fenofibrate found non-significant increases in adiponectin [20,21]. Therefore, in an attempt to replicate previous linkage results in Caucasians and to find novel areas of the genome linked to adiponectin, a whole-genome linkage scan of adiponectin measurements at baseline and after three-weeks of fenofibrate treatment was undertaken in the GOLDN Study.

## Methods

Detailed methods can be found in Additional File 1.

The study design and general population for the GOLDN Study have been previously described [22]. A total of 1121 individuals from 190 families had baseline adiponectin measurements and were included in the heritability estimates and linkage scans of baseline adiponectin. Of these, 859 individuals from 175 families completed the fenofibrate trial and had measurements of post-trial adiponectin and are thus included in analyses of post-trial adiponectin.

Plasma adiponectin was quantified using an ELISA assay from R & D Systems (Minneapolis, MN). Comparison of 58 blind replicates embedded in study samples showed the adiponectin assay had a reliability coefficient of 0.95. All participants were genotyped using the Marshfield Mammalian Genotyping Service screening set 12, which included 407 markers spaced at approximately every 10 cM across the genome. Heritability and linkage analyses were performed using SOLAR (Sequential Oligogenic Linkage Analysis Routines) [23]. All models were minimally adjusted for age, age^2^, sex, and field center. Some models were additionally adjusted for body mass index (BMI) in an attempt to reduce the proportion of variance in adiponectin due to environmental exposures. Two single-nucleotide polymorphisms (SNPs, rs17300539 and rs2241766) in the adiponectin gene (*ADIPOQ*) were genotyped using a TaqMan assay with allele-specific probes on the ABI PRISM 7900 HT Sequence Detection System (Applied Biosystems; Foster City, CA, USA) according to standardized laboratory protocols [24]. This study was approved by the centers' institutional review boards, and all subjects gave informed consent.

## Results

Table 1 shows the characteristics of all GOLDN participants included in these analyses and the subset of participants with both baseline and post-trial adiponectin measurements. On average the participants were in their late 40s, but there was substantial variability in the age of the study population (ages 18–87) as many families included two or three generations. On average, the participants were overweight (average BMI = 28.3 kg/m^2^). In the subset of the study population with baseline and post-trial adiponectin measurements, there was large variability in the change in adiponectin over the three-week trial of fenofibrate. The mean change in adiponectin was a decrease of 0.4 μg/ml (median decrease 0.3 μg/ml) with a standard deviation of 1.4 μg/ml. Changes over the three-week trial ranged from an increase of 5.8 μg/ml to a decrease of 6.2 μg/ml. Both transformed and non-transformed measurements of baseline and post-trial adiponectin (see Additional file 1) were highly correlated (r = 0.95, transformed measurements; r = 0.94, non-transformed measurements). Both baseline and post-trial adiponectin had similar correlations with BMI (r = -0.29 and r = -0.28, respectively).

**Table 1:** Characteristics (mean (standard deviation) or percentage) of the GOLDN Study sample

	**Participants with adiponectin measured at baseline (n = 1121)**	**Participants with adiponectin measured at both baseline and post-fenofibrate trial (n = 859)**
Age (years)	48.2 (16.3)	48.3 (15.9)
BMI (kg/m^2^)^†^	28.3 (5.6)	28.5 (5.5)
Baseline plasma adiponectin (μg/ml)*	7.4 (4.8 – 10.5)	7.2 (4.7 – 10.2)
Post-trial plasma adiponectin (μg/ml)*	--	7.0 (4.5 – 9.9)
Male (%)	48.1	49.6
*Center (%)*		
Minnesota	51.3	49.9
Utah	48.7	51.1

*median (interquartile range)

^†^measured at baseline

Table 2 presents the heritability estimates for both baseline and post-trial adiponectin. Heritability estimates increased somewhat after control for BMI. Heritability estimates also increased somewhat for post-trial measurements of adiponectin. However, heritability estimates for baseline adiponectin, calculated in the subset of individuals (n = 859) with post-trial measurements of adiponectin were nearly identical to those for post-trial adiponectin (data not shown).

**Table 2:** Heritability of baseline and post-fenofibrate trial adiponectin

Covariates*	**Heritability (SE)**	**p-value**	**Proportion of variance due to covariates**
*Baseline adiponectin*			
minimal model	0.38 (.06)	< .0001	.23
minimal model + BMI	0.44 (.06)	< .0001	.29
*Post-trial adiponectin*			
minimal model	0.46 (.07)	< .0001	.25
minimal model + BMI	0.55 (.07)	< .0001	.30

*Minimal model = age, age^2^, sex, field center

BMI, body mass index; SE, standard error

**Figure 1:**
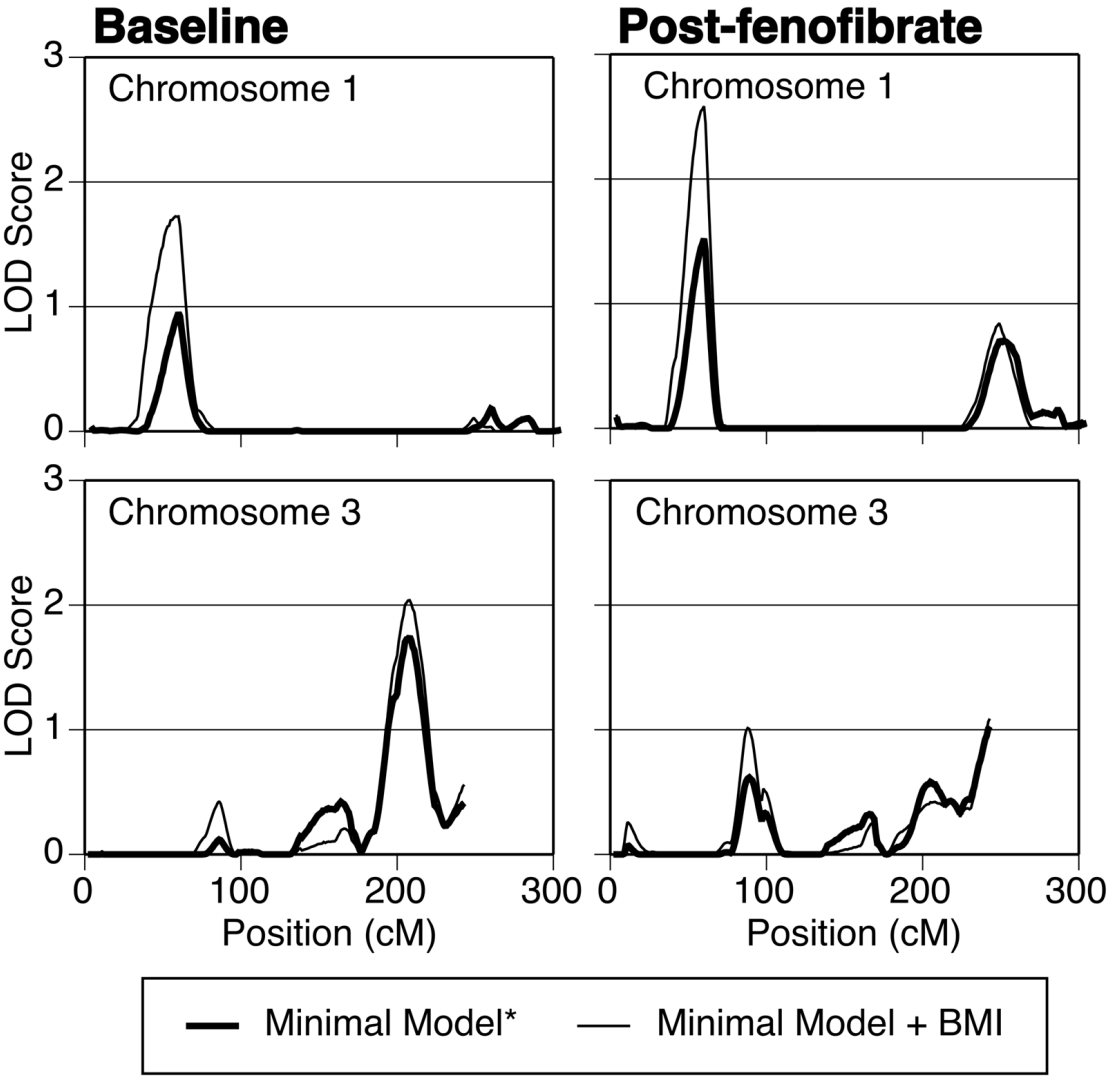
**Chromosomes 1 and 3 linkage results**. Chromosomes 1 and 3 linkage results for baseline and post-trial adiponectin. *Minimal model includes age, age^2^, sex, and field center.

Figure 1 shows the results of the linkage scans for baseline and post-trial adiponectin for chromosomes 1 and 3. Linkage scans for baseline adiponectin measured on the subset of individuals typed for post-trial adiponectin were nearly identical to the linkage scans for post-trial adiponectin and are thus not shown. Adjustment for BMI increased the height of the two highest linkage peaks (chromosomes 1 and 3) for both baseline and post-trial adiponectin. To assess whether association with two known polymorphisms in *ADIPOQ *explained the linkage on chromosome 3 (the location of *ADIPOQ*), additional genome scans were run adjusting for the two polymorphisms. Adjustment for *ADIPOQ *SNP genotypes (modeled additively) attenuated the peak on chromosome 3 only slightly; adjustment for SNP rs17300539 decreased the peak LOD from 2.04 to 1.91, adjustment for SNP rs2241766 decreased the peak LOD to 1.94, and adjustment for both SNPs decreased the peak LOD to 1.90.

Table 3 lists all the peaks with LOD scores over 1.5. The highest LOD score (2.58) was found on chromosome 1p35 for post-trial adiponectin (minimal model + BMI). The second highest LOD score (2.04) was found on chromosome 3q28 for baseline adiponectin (minimal model + BMI). For the peak on chromosome 1p35.2, the -1 LOD support interval was 50 cM – 64 cM (22.6 Mb-35.0 Mb) and this region contained 228 genes. Two candidate genes, interleukin 22 receptor, alpha 1 (*IL22RA1*, at 24.3 Mb) and interleukin 28 receptor, alpha (*IL28RA *at 24.4 Mb), were identified. For the peak on chromosome 3q28, the -1 LOD support interval was 195 cM -217 cM (180.8 Mb – 196.0 Mb) and this region contained 152 genes. One candidate gene, adiponectin (*ADIPOQ *at 188.0 Mb), was identified.

**Table 3:** Maximum LOD scores (over 1.5) for adiponectin

Covariates*	**Chromosome**	**Distance (cM)**	**LOD**	**p-value**	**Closest marker, location in bp^†^**
*Baseline adiponectin*					
minimal model + BMI	1p35.2	60	1.72	.0024	ATA79C10, 32.8 Mb
minimal model + BMI	3q28	208	2.04	.0011	D3S2398, 187.8 Mb
minimal model	3q28	208	1.75	.0023	D3S2398, 187.8 Mb
minimal model	6p23	26	1.60	.0033	D6S2434, 15.2 Mb
minimal model + BMI	7p14.2	54	1.81	.0019	D7S5817, 32.1 Mb
minimal model	15q22.32	62	1.80	.0020	D15S1507, 42.2 Mb
*Post-trial adiponectin*					
minimal model + BMI	1p35.2	60	2.58	.00028	ATA79C10, 32.8 Mb
minimal model	1p35.2	60	1.52	.0041	ATA79C10, 32.8 Mb

*minimal model = age, age^2^, sex, field center

^†^from the Celera map

BMI, body mass index; bp, base pair; LOD, logarithm of the odds

## Discussion

### Correlations of baseline adiponectin, post-trial adiponectin, and measures of adiposity

In the subset of the population (n = 859) where both baseline and post-trial adiponectin were measured, the correlation of the two adiponectin measurements was high (r = 0.95). On average, adiponectin decreased slightly over the course of the trial. GOLDN is the only trial we are aware of in which adiponectin concentrations fell following treatment with fenofibrate. Earlier trials either reported non-significant increases in adiponectin [20,21] or significant increases in adiponectin [18,19]. However, these studies were small (sample sizes ranging from 10 to 146) and used study populations with hypertriglyceridemia, severe obesity, or other chronic diseases. Given the small mean change in adiponectin and the very similar results for baseline and post-trial adiponectin heritability and linkage analyses when performed in the subset of individuals with both measures, the differences in analyses of baseline and post-trial adiponectin presented in this paper may be due to subtle differences in population structure between the full sample and subset.

Cross-sectional correlations between adiponectin and measures of adiposity have been reported in many other studies. The correlations between adiponectin and BMI in GOLDN were somewhat low, but still within the range of correlation values reported previously (r = -0.3 to -0.5) [4,25].

### Heritability analyses

The heritability of adiponectin has been examined previously in several large family studies [11-15]. The heritability estimates in the present study are consistent with results in other Caucasian study populations; the minimally-adjusted heritability estimate for baseline adiponectin (0.38) is similar to the unadjusted estimate from a similar sized study of northern Europeans [13] and the minimally adjusted post-trial adiponectin heritability estimate (0.46) is similar to an age and sex-adjusted estimate from a study conducted in the Old-Order Amish (0.55) [15]. Heritability estimates in GOLDN may be lower than those for some other study populations (particularly Hispanic or African American study populations) for a number of reasons. Different or additional genes may impact circulating adiponectin in these populations, or different environmental factors (such as obesity) may influence adiponectin in different populations.

### Linkage scans

No areas of (genome-wide) significant linkage were detected in this analysis. Two areas of suggestive linkage LOD > 2.0 were detected on chromosomes 1 and 3, and several areas of modest linkage (2.0 > LOD > 1.5) were also detected. Several genome-wide linkage scans of adiponectin have been published previously [12-16]. Comparing the results of these analyses to the results in the GOLDN Study reveals that the suggestive peak on chromosome 3q28, and the modest peak on 15q22.32 have been reported in other studies. In a study of Chinese siblings, a peak was found on chromosome 15 near the location of the peak detected in the present study [12]. Peaks around 200 cM on chromosome 3q have been reported previously in Hispanic families in the IRAS Family Study [14], in Northern European Families [13], and in the Old-Order Amish [15]. The adiponectin gene is located at 202 cM on chromosome 3, and many studies have established that variants in this gene influence adiponectin concentrations; a meta-analysis of these studies found a significant association between SNP rs17300539 and circulating adiponectin levels [26]. However, inclusion of this SNP and SNP rs2241766 (an *ADIPOQ *SNP shown to significantly attenuate an adiponectin linkage signal on chromosome 3 in an Amish population [15]) as covariates did not significantly attenuate the linkage signal in this study, indicating that other variants in *ADIPOQ *(or, potentially, variants in other genes under the linkage peak) may contribute to this linkage signal. This result mirrors the results of a linkage analysis in the IRAS Family Study were SNP rs17300539 was not found to be a major determinant of the linkage peak found on chromosome 3 [14].

The suggestive peak observed on chromosome 1p35.2 and the modest peaks observed on chromosomes 6p23 and 7p14.2 are novel. The peak observed on chromosome 1p35 in GOLDN has not been reported in other adiponectin linkage scans, but peaks have been found in that location in linkage scans of BMI and type 2 diabetes. Both the Quebec Family Study and a study in the Old-Order Amish detected significant areas of linkage (p = .05 and p = .0099) on chromosome 1p35.1 for BMI [27,28]. As the peak detected on chromosome 1 in GOLDN was not attenuated after adjustment for BMI (but rather increased after adjustment for BMI), it is unlikely that the linkage seen with adiponectin on chromosome 1 in GOLDN is due to a gene that indirectly affects adiponectin via direct effects on adiposity. Two possible explanations are that a pleiotropic gene lies under this peak which influences both adiposity and adiponectin concentrations through different pathways, or that two different genes (one influencing adiposity and another influencing adiponectin concentrations) both lie under this linkage peak. If either explanation is true, it is not surprising that multiple studies have also linked this region of the genome to type 2 diabetes [29,30], given the hypothesized relationships between adiposity, adiponectin, and insulin resistance.

There are several explanations for the novel modest linkages observed on chromosomes 6p23 and 7p14.2 in this study. It is possible that there are some genetic variants that impact circulating adiponectin in GOLDN, a Caucasian group of families from Utah and Minnesota, but not in other populations in which linkage scans have been conducted. It is also possible that some genes impact concentrations of circulating adiponectin in all populations, but that the effect of the genes was not detectable in some populations due to environmental variation, small effect sizes, and/or limited statistical power. In GOLDN, participants were not permitted to take lipid-lowering drugs or dietary supplements; this uniformity in the study population may have reduced environmental variation caused by these agents which was likely present in other linkage scans. Finally, given the modest size of the LOD scores for these linkages, these signals may represent false positive findings.

### Candidate genes

Identifying candidate genes for adiponectin is relatively difficult as little is known about upstream molecular regulators of adiponectin. Some studies have suggested that adiponectin production and secretion is regulated *in vivo *and *in vitro *by cytokines and their receptors such as IL-6 and TNF-α [31]. Another study has suggested that free fatty acids also acutely regulate adiponectin concentrations [32]. Finally, the cross-sectional association between adiponectin and adiposity is well established, so genes under the suggestive LOD peaks known to be involved with these three pathways were identified as possible candidate genes.

Two cytokine receptor genes were identified as candidate genes on chromosome 1p35. *IL22RA1 *encodes a portion of the receptor for interleukin 22. Interleukin 22 is a cytokine involved in the acute-phase inflammatory response. The second gene, *IL28RA*, also encodes a portion of a cytokine receptor; *IL28RA *is believed to be a subunit in the receptors for interleukin 28A, interleukin 28B, and interleukin 29. If interleukin 22, 28A, 28B, or 29 regulate adiponectin, then variations in genes involved in the receptors for these cytokines may impact circulating concentrations of adiponectin. The most likely explanation for the linkage peak observed on 3q28 is the adiponectin gene itself, as it has been shown that variation in this gene influences circulating concentrations of adiponectin in many populations. No other candidate genes were found under the peak on 3q28.

### Strengths and limitations

A primary strength of this study was that the study population likely had less variability in environmental exposures than other observational studies, as no individuals participating in the trial took lipid-lowering drugs or certain dietary supplements during the study period. Homogenous environmental exposures reduce the impact of gene by environment interactions which can mask linkage signals.

There were limitations to this study. The results may not be widely generalizable because of the homogeneity of the study population. Additionally, no household information was collected in the GOLDN Study which prevented the use of a household (shared environment) matrix in heritability and linkage analyses. As a result, heritability may have been overestimated if variance due to common environment was mistakenly attributed to variance due to additive genetic effects.

## Conclusion

This study performed whole-genome linkage scans for circulating adiponectin before and after a three-week trial of fenofibrate. There was a very high tracking correlation for adiponectin before and after the fenofibrate trial, and it was hypothesized that any meaningful difference in the heritabilities or linkage scans for the baseline and post-fenofibrate measurements of adiponectin was due to differences between the baseline sample and sub-sample that completed the trial of fenofibrate. Adiponectin was found to be moderately heritable, and the heritability increased after adjustment for BMI. No areas of significant (LOD > 3) linkage were discovered, but there were two suggestive (LOD > 2) peaks on chromosomes 1p35.2, and 3q28. The peak on chromosome 1 corroborates areas of significant linkage for BMI and type 2 diabetes mellitus. The peak on chromosome 3 contains *ADIPOQ*.

## Abbreviations

BMI: body mass index; GOLDN: The Genetics of Lipid Lowering Drugs and Diet Network; LOD: logarithm of the odds.

## Competing interests

The authors declare that they have no competing interests.

## Authors' contributions

LJR-T analyzed and interpreted the data and drafted the manuscript. JSP helped in the analysis and interpretation of the data. JSP, JMP, IBB, JEH, MYT, EKK, and DKA were involved in the design of the study and the acquisition and preparation of genetic and phenotypic data used for this manuscript. All authors participated in critical revisions of the manuscript and all have read and approved the final manuscript.

## Pre-publication history

The pre-publication history for this paper can be accessed here:



## Supplementary Material

Additional File 1 **Detailed Materials and Methods**. The text provides details on the design of the GOLDN study and additional description of the methods used in this analysis. Click here for file
