# Supplementary material for: Suggestion for linkage of chromosome 1p35.2 and 3q28 to plasma adiponectin concentrations in the GOLDN Study
Source: BMC Med Genet. 2009 May 9;10:39. doi: 10.1186/1471-2350-10-39 (PMC2691741; doi:10.1186/1471-2350-10-39)
Supplement: Additional File 1 — Detailed Materials and Methods. The text provides details on the design of the GOLDN study and additional description of the methods used in this analysis. [file 1471-2350-10-39-S1.doc]

*Additional file 1*

**Suggestion for linkage of chromosome 1p35.2 and 3q28 to plasma adiponectin concentrations in the GOLDN Study**

Laura J. Rasmussen-Torvik, James S. Pankow, James M. Peacock, Ingrid B. Borecki, James E. Hixson, Michael Y. Tsai, Edmond K. Kabagambe, Donna K. Arnett

**Detailed Materials and Methods**

*Study design*

The GOLDN study examined expression of genes involved in lipid and fat metabolism in two different extreme environments i.e., under treatment with fenofibrate and after a dietary fat challenge, as has been discussed in detail elsewhere [1]. As no variables included in these analyses were collected during the dietary fat challenge, this aspect of this study is not discussed. Baseline visits were scheduled 28 days after participants discontinued use of lipid-lowering medications. At baseline visits, informed consent was obtained, blood for DNA analysis and adiponectin measurements was drawn, and anthropometric measurements were obtained. Individuals were given a daily prescription for fenofibrate (160 mg daily, to be taken with the morning meal). After three weeks of daily treatment with fenofibrate, the participants returned for post-trial measurement of adiponectin as well as other variables such as lipids and anthropometrics.

*Study Population*

Recruitment for the GOLDN study has been described in detail elsewhere [1]. Briefly, the largest three-generation families were recruited from the pool of families that had participated in the National Heart, Lung, and Blood Institute Family Heart Study (FHS) at either the Minnesota or Utah field centers. To expand pedigrees, additional offspring who had not participated in FHS were recruited to participate in GOLDN, and three de novo families were recruited in Minnesota. Only family members over the age of 18 were allowed to participate. Individuals were excluded from the study if they were pregnant, not using birth control, or nursing; had a myocardial infarction, coronary artery bypass, or coronary angiography in the past 6 months; used insulin; had an abnormal glomerular filtration rate (< 30mL/min/1.73m^2^); had extremely elevated plasma triglycerides (> 1500mg/dl), aspartate aminotransferase ( > 42U/L), or alanine aminotransferase ( > 44U/L [males], > 66U/L [females] ); or were unwilling or unable to cease taking lipid-lowering drugs or dietary supplements for four weeks before and during the study. In addition, participants with a known sensitivity to fenofibrate were excluded.

A total of 1121 individuals from 190 families had baseline adiponectin measurements and were included in the heritability estimates and linkage scans of baseline adiponectin. Of these, 859 individuals from 175 families completed the fenofibrate trial and had measurements of post-trial adiponectin and are thus included in analyses of post-trial adiponectin.

*Phenotypes*

Participants were asked to fast for at least 12 hours and abstain from alcohol for at least 24 hours before each study visit. Weight was taken with minimal clothing on a balance. Results were recorded to the nearest pound, rounding down. Height was measured with the participant standing as straight as possible without shoes with feet flat on the floor. The height was recorded to the nearest centimeter, rounding down to the nearest centimeter if the measurement fell between centimeter marks. Plasma adiponectin was measured in the Collaborative Studies Laboratory at the University of Minnesota Medical Center, Fairview. Plasma adiponectin was quantified using an ELISA assay from R & D Systems (Minneapolis, MN). Comparison of 58 blind replicates embedded in study samples showed the adiponectin assay had a reliability coefficient of 0.95.

*Genotypes*

DNA extraction and purification were performed at University of Minnesota Medical Center, Fairview using a standard protocol. Microsatellite genotyping used fluorescently labeled PCR primers for marker amplification followed by capillary electrophoresis on automated DNA sequencers (3730xl DNA Analyzer, Applied Biosystems, Inc (ABI), Foster City, CA) at the University of Texas-Houston Human Genetics Center. All participants were genotyped using the Marshfield Mammalian Genotyping Service screening set 12 which included 407 markers spaced at approximately every 10 cM across the genome.

Genotypes were assigned using GeneMapper® software from ABI and tested for consistency with known relationships among GOLDN pedigrees using the computer program GRR (Graphical Representation of Relationships by G.R. Abecasis). Mendelian errors were detected and zeroed out using the computer program Pedstats [2].

*Statistical Analysis*

Means, medians, and correlations of trait variables were estimated using SAS version 9.0. Both baseline and post-trial adiponectin were highly skewed. Variance components statistical methods are not robust to non-normality [3] so baseline adiponectin and post-trial adiponectin were forced into a normal distribution using the Blom transformation [4] as implemented in SAS proc rank. Transformed adiponectin values were used in all variance components and correlation analyses.

Heritability and linkage scans were performed using variance components methods. For heritability analyses the phenotypic trait variance was partitioned into variance attributable to additive genetic effects and environmental effects. Additive genetic variance was modeled as a function of expected covariances between relatives. For linkage scans the additive genetic variance was further partitioned into quantitative trait locus (QTL) variance and residual genetic variance with the QTL variance being modeled as a function of the identity-by descent probabilities (IBDs) at each marker locus and the residual genetic variance being modeled as a function of the expected covariances between relatives. IBDs were calculated using the computer program LOKI [5] with allele frequencies estimated from the study population. The presence of linkage was tested using a likelihood ratio test comparing a model where the QTL variance was freely estimated to a model where the QTL variance was constrained to be zero [6].

Heritability and linkage analyses were performed using SOLAR (Sequential Oligogenic Linkage Analysis Routines) [6]. Possible covariates were tested for significance using the polygenic screen option. Only significant covariates were included in analyses with the exception of field center, which was included (although it did not account for a significant portion of the variance) to account for any measurement differences between study sites*.* Therefore, all models were minimally adjusted for age, age^2^, sex, and field center. Some models were additionally adjusted for BMI, in an attempt to reduce the proportion of variance in adiponectin due to environmental exposures. Only one individual in the study indicated no white ancestry, and this individual was excluded from linkage scan analyses. Candidate genes were examined under suggestive linkage peaks (LOD > 2). Candidate genes were identified by searching for genes with possible relationships to adiponectin within the NCBI Map Viewer database using borders defined by the Marshfield map. The region examined was determined by the 1 LOD score support interval around the highest peak in that region.

**References**

1. Lai CQ, Arnett DK, Corella D, Straka RJ, Tsai MY, Peacock JM, Adiconis X, Parnell LD, Hixson JE, Province MA *et al*: **Fenofibrate effect on triglyceride and postprandial response of apolipoprotein A5 variants: the GOLDN study**. *Arterioscler Thromb Vasc Biol* 2007, **27**(6):1417-1425.

2. Abecasis GR, Cherny SS, Cookson WO, Cardon LR: **Merlin--rapid analysis of dense genetic maps using sparse gene flow trees**. *Nat Genet* 2002, **30**(1):97-101.

3. Amos CI, de Andrade M: **Genetic linkage methods for quantitative traits**. *Stat Methods Med Res* 2001, **10**(1):3-25.

4. Blom G: **Statistical elements of transformed beta variables**. New York: Wiley; 1958.

5. Heath SC: **Markov chain Monte Carlo segregation and linkage analysis for oligogenic models**. *Am J Hum Genet* 1997, **61**(3):748-760.

6. Almasy L, Blangero J: **Multipoint quantitative-trait linkage analysis in general pedigrees**. *Am J Hum Genet* 1998, **62**(5):1198-1211.
